# Study of Cardiac Dysfunction in Portal Hypertension: A Single-Center Experience From Eastern India

**DOI:** 10.7759/cureus.51259

**Published:** 2023-12-28

**Authors:** Sambit Kumar Behera, Prajyoti Behera, Jyoti Ranjan Behera, Gayatri Behera

**Affiliations:** 1 Gastroenterology, Srirama Chandra Bhanja (SCB) Medical College, Cuttack, IND; 2 Physiology, Institute of Medical Sciences (IMS) & SUM Hospital, Bhubaneswar, IND; 3 Pediatrics, Kalinga Institute of Medical Sciences, Bhubaneswar, IND; 4 Pathology, Institute of Medical Sciences (IMS) & SUM Hospital, Bhubaneswar, IND

**Keywords:** ehpvo, budd-chiari syndrome, liver disorder, cirrhosis, cardiac dysfunction

## Abstract

Introduction: Cardiac functional abnormalities are common in patients with cirrhosis of the liver. Nonetheless, the effect of portal hypertension and liver disorder on cardiac abnormalities is yet to be investigated. The current study evaluated the contribution of cirrhotic and non-cirrhotic portal hypertension as the potential cause of cardiac abnormalities.

Methods: The present study was a cross-sectional observational study. After excluding known heart diseases, 128 patients with portal hypertension from different causes were enrolled in the study. Cardiac functional activity was assessed by electrocardiogram (ECG) and transthoracic echocardiography (TTE).

Results: This study included a total of 128 patients, out of which 24 had extrahepatic portal vein obstruction (EHPVO), four patients had Budd-Chiari syndrome and 100 had liver cirrhosis. Normal ventricular function was observed in patients with EHPVO and Budd-Chiari syndrome. Sixty-eight percent of cases had liver cirrhosis diastolic abnormalities. The mean QTc interval in patients with cirrhotic cardiomyopathy (CCM) was 0.49 ± 0.05 sec which was significantly increased when compared to patients without CCM with 0.432 ± 0.07 at *p*=0.0016. The Child Turcotte Pugh (CTP) score and MELD (Model for End-Stage Liver Disease) score in patients with CCM were significantly higher as compared to patients without CCM. All alcoholic cirrhotic and non-alcoholic cirrhotic patients had equal prevalence of diastolic dysfunction (*p*-value >0.05).

Conclusion: Patients with Child class C or a high MELD score are associated with a higher prevalence rate of CCM while normal cardiac function was observed among patients having portal hypertension due to extrahepatic causes. We recommend cardiac evaluation by echocardiography in all cirrhotic patients. Institution of specific medical therapy and early referral for liver transplantation should be considered to improve survival in patients with decompensated cirrhosis.

## Introduction

Cirrhotic cardiomyopathy (CCM) is defined as a long-standing cardiac dysfunction in cirrhotic patients pertaining to decreased contractile response to stress and/or troubled diastolic relaxation with electrophysiological aberrations without any known cardiac disease [1]. Moreover, heart failure is not a striking component in cirrhosis of the liver; however, the presence of latent cardiac dysfunction is an established component [1,2].

In cirrhotic patients, ventricular function is impaired in response to stress (physiological or drug-induced) [3,4]. Heart failure appeared as a significant reason for death after orthotopic liver transplantation, accounting for 7-21% of deaths in the post-transplantation period [5]. Diastolic dysfunction, which occurs before systolic dysfunction at rest, is shown to be an early marker of cardiac dysfunction [6]. The mechanical barrier to ventricular function could account for the diastolic dysfunction in the presence of ascites [2,7,8]. Collection of cardio-depressant materials as a result of hepatocellular failure had been proposed as one of the feasible causes of cardiac changes in cirrhotic patients as revealed from a previous study [1].

The relative contribution of portal hypertension and hepatic dysfunction in liver cirrhosis is unclear. The current study aimed to evaluate portal hypertension with and without hepatic dysfunction to discern the role of portal hypertension and cirrhosis in causing cardiac changes.

## Materials and methods

The study protocol was approved by the Institutional Ethics Committee. This study included 128 patients with the diagnosis of portal hypertension from different causes. Twenty-five normal subjects were taken for comparison with the study population.

All suspected/confirmed cases of portal hypertension irrespective of the cause were included in the study. Patients with a past history of cardiac diseases like myocardial infarction, valvular heart disease, various conduction abnormalities, heart failure, essential hypertension, dyselectrolytemia, history of drug intake like calcium channel blockers, digoxin, antiarrhythmics, and digoxin were excluded from the study.

Portal hypertension was confirmed by upper gastrointestinal endoscopy and imaging study. Upper gastrointestinal endoscopy displayed esophageal varices, fundal varices, and portal hypertensive gastropathy. Transabdominal ultrasound showed portal vein diameter >12 mm with intra-abdominal collaterals.

Laboratory testing included hemoglobin (g/dL), differential count, total leukocyte count, total platelet count, blood sugar (mg/dL), urine (routine/microscopy), stool (routine/microscopy), liver function test (U/L), serum albumin (g/dL), and protein, different coagulation tests, blood urea nitrogen (mg/dL), creatinine (mg/dL), serum sodium and potassium (mmol/L), viral serology, particularly hepatitis B and C, laboratory tests (special cases), antinuclear antibody, smooth muscle antibody and anti LKM antibody (where indicated), anti-mitochondrial antibody (if indicated) and iron indices including serum ferritin (ng/mL), transferrin saturation (%) in suspected cases of hemochromatosis, alpha1-antitrypsin deficiency (if indicated), ceruloplasmin (mg/dL), 24-hour urinary copper, and Kayser-Fleischer ring in cases suspected of Wilsons disease.

The severity of cirrhosis was evaluated by the Child-Turcotte-Pugh (CTP) score and MELD (Model for End-Stage Liver Disease) score. A fibro-scan was done to assess liver stiffness; the resting of ECG (electrocardiogram) in all patients was done in the medical unit by a certified ECG technician QTc (corrected for heart rate) values were obtained in the patients by the formulae QTc= QT/sq root RR. The value of QTc > 0.44 sec was regarded as prolonged; heart rate was obtained via the formula HR = 1500 / RR. The existence of prolonged QTc and heart rate >100 was labeled as abnormalized ECG. An echocardiographic examination was carried out by a consultant cardiologist who did not know the primary diagnosis, including a two-dimensional echo and color flow Doppler study. Systolic dysfunction was assessed as ejection fraction value <55% was considered as decreased. Moreover, the diastolic dysfunction substantiated by the abnormal mitral E/A ratio {early (E) to late (A) ventricular filling velocities} was estimated.

CCM was diagnosed if the structural and functional ventricular abnormalities in echocardiographic studies are left ventricular hypertrophy and evidence of either systolic or diastolic dysfunction or an abnormal ventricular response in the presence of pharmacologic, physiologic, or surgical stress or cardiac electrophysiologic abnormalities especially prolongation of the corrected QT interval (QTc).

Statistical analysis

The age (year), QTc interval (sec), and E/A ratio were all expressed as mean ± SD. Categorical variables like gender, Child-Pugh classification, increased E/A ratio, prolonged QT interval, ejection fraction (EF), and the absence or presence of CCM were expressed as freq(%). The Chi-square/ Fisher’s exact test was used to check the association of categorical variables with CCM, like Child-Pugh Class, E/A ratio less than 1 or equal to or greater than 1, QT interval less than 0.44 sec or equal to or greater than 0.44 sec, and EF less than 55% or equal to or greater than 55%. A p-value < 0.05 was considered statistically significant. For all calculations, IBM SPSS Statistics for Windows, Version 16 (Released 2007; IBM Corp., Armonk, New York, United States) was used.

## Results

A total of 128 patients from January 2016 to January 2018 were enrolled in the study; 25 healthy individuals as matched controls were also taken for the comparison. Males constituted 66% of cases and the rest were female individuals; moreover, among the healthy control participants 60% were males. The mean age of the patients was 44.86 ± 15.90 years, and the mean age of the controls was 48.6 ± 18.06 years (p=0.3619). The mean serum bilirubin in the patients was 6.43± 5.48 mg/dl which was significantly higher than the controls in which it was 0.91 ± 0.147 mg/dl (p=0.0007). Similarly, ALT (alanine aminotransferase) and AST (aspartate aminotransferase) in the patients were significantly higher as compared to the controls. The serum albumin in the control was 3.59 ± 0.56 g/dl and in the patients was 3.05±0.731 g/dl which was significantly lower (p=0.0018). The prothrombin time and INR (International normalized ratio) were also significantly raised in patients as compared to the controls (Table 1).

**Table 1:** Demographic profile and serum biochemistry of patients and controls ALT: Alanine aminotransferase, AST: aspartate aminotransferase, PT: prothrombin time, INR: International normalized ratio.

Total number	Patients	Controls	p-value
	128	25	
M/F	85/43 66/34 %	15/10 60/40 %	
Age (year) (mean ± SD)	44.86 ± 15.90	48.6 ± 18.06	0.3619
Serum Bilirubin (mg/dl) (mean ± SD)	6.43 ± 5.48	0.91 ± 0.147	0.0007
ALT (U/L) (mean ± SD)	153.99 ± 89.58	38.52 ± 24.875	0.0310
AST (U/L) (mean ± SD)	149.08 ± 113.47	36.48 ± 15.311	0.0006
Serum albumin (g/dl) (mean ± SD)	3.05±0.731	3.59 ± 0.56	0.0018
Serum creatinine (mg/dl) (mean ± SD)	1.23 ± 0.311	0.99 ± 0.28	0.0925
PT (srcond) (mean ± SD)	20.60± 8.458	14.88±8.42	0.0072
INR (mean ± SD)	1.77± 0.828	1.27 ± 0.073	0.0037

The most common cause of portal hypertension was cirrhosis of the liver which was found in 78.12% of patients. It was followed by EHPVO (extrahepatic portal vein obstruction) and Budd-Chiari syndrome (18.75 and 3.12% respectively).

The most common cause of cirrhosis was alcohol which was responsible for cirrhosis in 41.4% of patients with hypertension. It was followed by cryptogenic and hepatitis cirrhosis (15.6 and 13.28% respectively). NASH-related cirrhosis of the liver was seen in 7.82% of patients. The most common presenting symptom was abdominal distension, present in 90 (70.31%) patients followed by hematemesis/melena in 86 (67.18%) patients, and altered sensorium in 8 (6.25%) patients.

**Figure 1:**
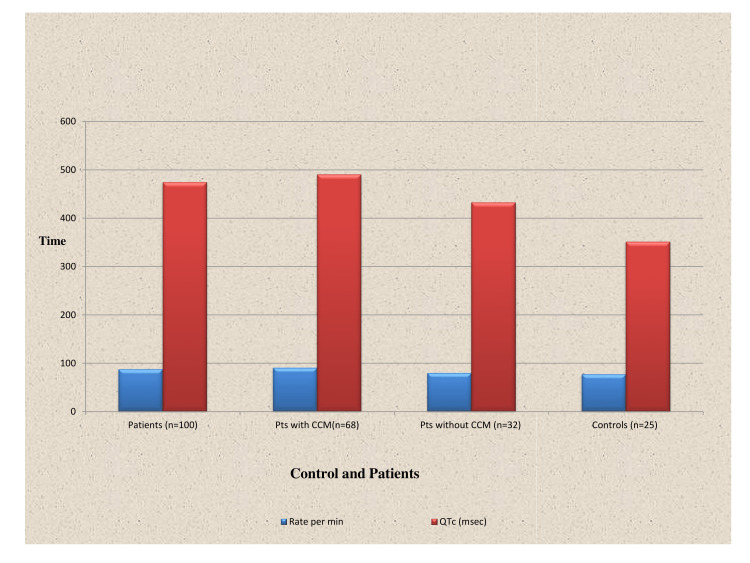
Child’s score and MELD score in cirrhotics CCM: Cirrhotic cardiomyopathy, MELD: Model for End-Stage Liver Disease, QTc: corrected for heart rate

The patients with EHPVO and Budd-Chiari syndrome had normal echocardiography showing no evidence of systolic or diastolic dysfunction. In patients with cirrhosis of the liver (n=100), 68% of patients had diastolic dysfunction. Fourteen percent of patients belonged to Child class A, 48 % to Child class B, and 38% to Child class C. The mean CTP score in patients was 8.94 ± 2.06. The mean CTP score in patients with CCM was 9.65 ± 1.97 while in patients without CCM it was 7.44± 1.24 and this difference in the CTP score was statistically significant (p=0.0002) (Table 2 and Figure 1). The mean MELD score in the patients was 17.26 ± 13.65. The mean MELD score in patients with CCM was 18.97 ± 7.17 while in patients without CCM it was 13.625 ± 6.18 and this difference in the MELD score was statistically significant (p=0.0135) (Table 2 and Figure 1). 

**Table 2:** Child’s status and MELD score in cirrhotics CTP: Child-Turcotte-Pugh, MELD: Model for End-Stage Liver Disease.  Statistics were calculated at 95% confidence interval

Child class A	14(14 %)
Child class B	48 (48%)
Child class C	38 (38%)
CTP score in patients (n=100)	8.94 ± 2.06
CTP score in patients with cirrhotic cardiomyopathy (n=68)	9.65 ± 1.97
CTP score in patients without cirrhotic cardiomyopathy (n=32)	7.44± 1.24 (p=0.0002)
MELD score in all patients (n=100) (mean ± SD)	17.26 ± 13.65
MELD score in patients with cirrhotic cardiomyopathy (n=68)	18.97 ± 7.17
MELD score in patients without cirrhotic cardiomyopathy (n=32)	13.625 ± 6.18 (p=0.0135)

Out of 68 patients with CCM, 4 belonged to Child class A, 28 to Child class B, and 36 to Child class C. Out of 32 patients without CCM, 10 belonged to Child class A, 20 to Child class B, and 2 to Child class C. Similarly, out of 14 Child class A patients, four had CCM and 10 didn’t; out of 48 Child class B patients 28 had CCM and 20 didn’t; out of 38 Child class C patients 36 had CCM and 2 didn’t. The calculated p-value was <0.001 indicating that as the severity of cirrhosis increases, CCM becomes more prevalent (Table 3).

**Table 3:** Child’s category and cirrhotic cardiomyopathy

	Child A (n=14)	Child B (n=48)	Child C (n=38)	p-value
Cirrhotic Cardiomyopathy present (n=68)	4 (28.57 %)	28 (58.33%)	36 (94.73%)	<0.001
Cirrhotic Cardiomyopathy absent (n=32)	10 (71.42%)	20 (41.66%)	2 (5.26%)	

**Figure 2:**
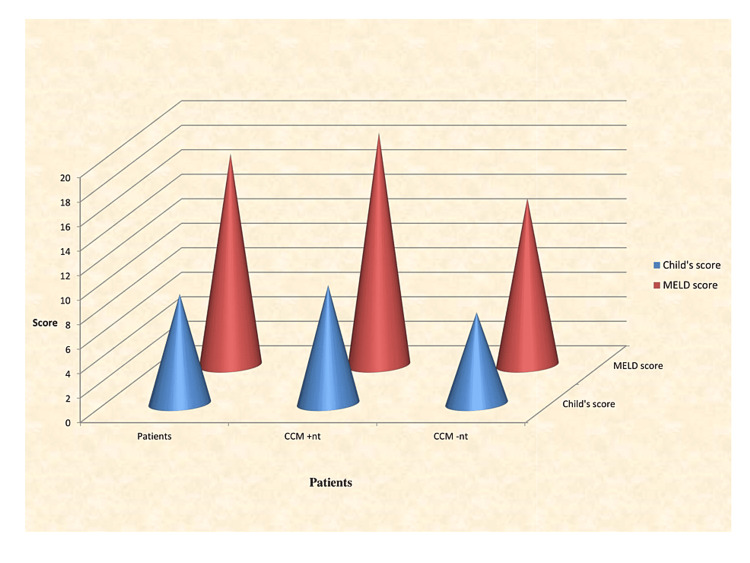
Comparison of electrocardiography alterations in patients and controls CCM: Cirrhotic cardiomyopathy, MELD: Model for End-Stage Liver Disease

The mean heart rate in the patients was 86.66 ± 13.08 /min which was significantly higher as compared to the controls in which it was 76.32± 13.166/min (p=0.0019). The mean heart rate in patients with CCM was 90.18 ± 11.58/min which was significantly higher as compared to patients without CCM in which it was 79.19 ± 13.07/min (p=0.0042) (Table 4). The QTc interval in the patients was 0.474 ± 0.066 sec which was significantly increased as compared to the controls in which it was 0.351±0.055 (p=0.0001). The mean QTc interval in patients with CCM was 0.49 ± 0.05 sec which was significantly increased as compared to patients without CCM in which it was 0.432 ± 0.07(p=0.0016) (Table 4 and Figure 2). 

**Table 4:** Comparison of electrocardiography alterations in patients and controls CCM: Cirrhotic cardiomyopathy, QTc: corrected for heart rate. Statistics were calculated at 95% confidence interval

	Patients (n=100)	Patients with CCM (n=68)	Patients without CCM (n=32)	p-value
Heart Rate (/min)	86.66 ± 13.08	90.18 ± 11.58	79.19 ± 13.07	<0.001
QTc (seconds)	0.474 ± 0.066	0.49 ± 0.05	0.432 ± 0.07	<0.001

The basal EF and E/A ratio in the patients was significantly lower while basal ESV was significantly higher in patients with cirrhosis as compared to the controls. Basal EDV was not significantly different between the two groups (Table 5). The basal E/A ratio was significantly lower in patients with CCM as compared to patients without CCM while basal EF, EDV, and ESV were not significantly different between the two groups (Table 5).

**Table 5:** Comparison of echocardiographic findings between patients and controls EF: Ejection fraction, EDV: end diastolic volume, ESV: end systolic volume, E/A: early (E) to late (A) ventricular filling velocities. Statistics were calculated at 95% confidence interval

	Controls (n=25)	Patients without cirrhosis (n=28)	Patients with cirrhosis (n=100)	Cirrhotic cardiomyopathy present(n=68)	Cirrhotic cardiomyopathy absent(n=32)
EF (%)	60.93±3.84	60.12±3.24	57.28±6.02 (p=0.0073)	56.41±6.7	59.14±4.16 (p=0.1414)
EDV (ml)	84.28±7.18	83.76±8.11	83.06±8.15 (p=0.5275)	82.91±8.19	83.38±8.57 (p=0.8528)
ESV (ml)	33.2±2.58	33.43±2.41	35.28±4.81 (p=0.0473)	35.97±5.57	33.81±2.3 (p=0.143)
E/A	1.096±0.08	1.12±0.04	0.89±0.22 (p=0.0001)	0.785±0.16	1.11±0.19 (p < 0.05)

Cardiac dysfunction was equally prevalent among patients with cirrhosis due to alcohol and other etiologies (p-value >0.05) (Table 6).

**Table 6:** Comparison of echocardiographic findings in different types of cirrhosis EF: Ejection fraction, EDV: end diastolic volume, ESV: end systolic volume, E/A: early (E) to late (A) ventricular filling velocities. Statistics were calculated at 95% confidence interval.

	Controls (n=25)	Alcoholic cirrhosis (n=53)	Cryptogenic cirrhosis (n=20)	Hepatitis-B-related cirrhosis (n=17)	NASH-related cirrhosis (n=10)	p-value
EF (%)	60.93±3.84	56.55±5.8	57.41±6.8	57.33±6.5	56.23±5.2	0.1025
EDV (ml)	84.28±7.18	82.90±7.33	83.94±7.52	82.98±7.16	82.88±8.04	0.4472
ESV (ml)	33.2±2.58	36.97±4.42	35.88±4.58	35.84±4.59	36.98±5.34	0.0602
E/A	1.096±0.08	0.744±0.24	0.814±0.26	0.822±0.56	0.732±0.54	0.0832

## Discussion

CCM refers to a group of symptoms that indicate an abnormal heart structure and function in cirrhotic patients. Systolic and diastolic dysfunction, electrophysiological changes, and macroscopic and microscopic structural changes were observed in the study. Functional changes were frequently accompanied by a significant stress challenge such as exercise or pharmacological [9]. The exact prevalence of CCM is unknown due to a lack of clear diagnostic criteria. It is difficult to estimate because the disease is generally latent and only manifests itself once the patient is exposed to stressors like exercise, drugs, and hemorrhage. The prevalence of liver cirrhosis is also difficult to estimate as compensated cirrhosis usually does not show symptoms of the disease and non-invasive studies usually lack sensitivity to detect cirrhosis in its early stages. CCM was found in 68% of cirrhotic patients in the current study. It was previously reported that the prevalence of CCM was 44.6%, which was lower than in the current study [10].

The prevalence of CCM significantly increased; it was present in patients with Child's class A, B, and C cirrhosis in 28.57%, 58.33%, and 94.74% of patients, respectively. The prevalence of CCM increased from 25% in Child class A to 51% in Child class B to 60% in Child class C in a previous study [11]. Additionally, as the cirrhosis progressed in severity, the frequency of CCM increased proportionally in another study [12]. Moreover, in the current study, CCM was significantly higher in CTP scores and MELD scores than patients without CCM.

The mean heart rate in the patients was significantly increased as compared to the controls in the current study which was corroborated with the other studies [10,13]. Furthermore, the mean heart rate in patients with CCM was significantly increased as compared to patients without CCM. In another study, it was observed that cirrhosis advanced the hyperdynamic circulation characterized by tachycardia, increased cardiac output, and high ejection fraction [14]. The electrophysiological abnormalities in CCM included prolonged repolarization, which manifested itself in the form of prolonged QT interval. In the present study, QTc in the patients was significantly increased as compared to the controls. The mean QTc in patients with CCM was significantly increased as compared to the patients without CCM. Increased QTc was seen in 74% of patients in the current study, and QTc>0.44 sec was strongly associated with the presence of CCM. A prolonged QTc was found in 45% of patients [15] which is slightly lower than the present study. A much lower frequency of prolonged QTc was found in the previous study, suggestive of 19.2% of cirrhotic patients who had a prolonged QTc [16].

Diastolic dysfunction was manifested by a reversed E/A ratio (E/A<1); in the current study, a fraction of 68% of patients exhibited these abnormalities. This corroborated with the other study which reported that around 50% of patients had E/A ratio reversal at rest, especially when decompensation occurs with ascites due to cirrhosis [17]. When paracentesis was performed, a significant improvement was found in the E/A ratio, which was not reverted like that of a normal person but it improved significantly [18]. Moreover, another study highlighted on presence of diastolic dysfunction in a greater number of patients with cirrhosis [19]. Diastolic dysfunction was very common in old age; certainly, few studies insist that mild diastolic dysfunction was observed in almost all patients suffering from cirrhosis [14,20-22]. Hence, assessment of diastolic dysfunction with only TTE was not sufficient to demarcate true CCM patients from general cirrhotic patients. The most outstanding property of CCM was LV systolic dysfunction compared with general cirrhotic patients. Therefore, a provocative test to measure systolic dysfunction of the heart in patients with liver cirrhosis based on CCM pathophysiology was required as a screening test. So far, multiple experiments have been made to stress the ventricle by different physiological or pharmacological methods. In the case of the exercise test, the provocation test is difficult to perform in the case of cirrhotic patients due to reduced exercise capacity because of the aging process or other causes unrelated to cardiac function [23,24].

Limitations

Ideally, all subjects should have been subjected to stress tests, especially the dobutamine stress test, but in our study, it couldn’t be performed due to logistical constraints. Follow-up is required to look for adverse cardiac events and outcomes in patients with CCM diagnosed on the basis of echocardiographic and ECG abnormalities as was performed in this study. This is necessary to ascertain the significance of this entity and the need to identify them. This was not performed in our study. Although we did not find any changes in ECG and echocardiography in patients with non-cirrhotic portal hypertension, the number of these patients in our study was low. There is a need to look for changes in cardiomyopathy with a larger number of noncirrhotic patients.

## Conclusions

In conclusion, we found CCM in more than two-thirds of hospitalized cirrhotic patients. With increasing severity of hepatic dysfunction, the prevalence of CCM has also increased. Patients with Child class C or a high MELD score are associated with a higher prevalence rate of CCM. In contrast, cardiac function is normal among patients with portal hypertension due to extrahepatic causes. Therefore, cardiac evaluation by echocardiography should be done in all cirrhotic patients. Institution of specific medical therapy and early referral for liver transplantation should be considered to improve survival in patients with decompensated cirrhosis.

## References

[1] Pozzi M, Ratti L, Guidi C, Milanese M, Mancia G (2007). Potential therapeutic targets in cirrhotic cardiomyopathy. Cardiovasc Hematol Disord Drug Targets.

[2] Liu H, Ma Z, Lee SS (2000). Contribution of nitric oxide to the pathogenesis of cirrhotic cardiomyopathy in bile duct-ligated rats. Gastroenterology.

[3] La Villa G, Romanelli RG, Casini Raggi V (1992). Plasma levels of brain natriuretic peptide in patients with cirrhosis. Hepatology.

[4] Wong F, Siu S, Liu P, Blendis LM (2001). Brain natriuretic peptide: is it a predictor of cardiomyopathy in cirrhosis?. Clin Sci (Lond.

[5] Girgrah N, Reid G, MacKenzie S, Wong F (2003). Cirrhotic cardiomyopathy: does it contribute to chronic fatigue and decreased health-related quality of life in cirrhosis?. Can J Gastroenterol.

[6] Ginès P, Uriz J, Calahorra B (2002). Transjugular intrahepatic portosystemic shunting versus paracentesis plus albumin for refractory ascites in cirrhosis. Gastroenterology.

[7] KO HJ, AB WH (1953). The cardiac output at rest in Laennec's cirrhosis. J Clin Invest.

[8] Rayes N, Bechstein WO, Keck H, Blumhardt G, Lohmann R, Neuhaus P (1995). Cause of death after liver transplantation: an analysis of 41 cases in 382 patients (Article in German). Zentralbl Chir.

[9] Møller S, Henriksen JH (2002). Cirrhotic cardiomyopathy: a pathophysiological review of circulatory dysfunction in liver disease. Heart.

[10] Shaikh S, Abro M, Qazi I, Yousfani A (2011). Frequency of cirrhotic cardiomyopathy in patients with cirrhosis of liver: a tertiary care hospital experience. Pak J Med Sci.

[11] Bernardi M, Calandra S, Colantoni A (1998). Q-T interval prolongation in cirrhosis: prevalence, relationship with severity, and etiology of the disease and possible pathogenetic factors. Hepatology.

[12] Yildiz R, Yildirim B, Karincaoglu M, Harputluoglu M, Hilmioglu F (2005). Brain natriuretic peptide and severity of disease in non-alcoholic cirrhotic patients. J Gastroenterol Hepatol.

[13] Ma Z, Lee SS (1996). Cirrhotic cardiomyopathy: getting to the heart of the matter. Hepatology.

[14] Gaskari SA, Honar H, Lee SS (2006). Therapy insight: cirrhotic cardiomyopathy. Nat Clin Pract Gastroenterol Hepatol.

[15] Clark JM (2006). The epidemiology of nonalcoholic fatty liver disease in adults. J Clin Gastroenterol.

[16] Zuberi BF, Ahmed S, Faisal N (2007). Comparison of heart rate and QTc duration in patients of cirrhosis of liver with non-cirrhotic controls. J Coll Physicians Surg Pak.

[17] Pozzi M, Ratti L, Redaelli E, Guidi C, Mancia G (2006). Cardiovascular abnormalities in special conditions of advanced cirrhosis. The circulatory adaptative changes to specific therapeutic procedures for the management of refractory ascites. Gastroenterol Hepatol.

[18] Henriksen JH, Gøtze JP, Fuglsang S, Christensen E, Bendtsen F, Møller S (2003). Increased circulating pro-brain natriuretic peptide (proBNP) and brain natriuretic peptide (BNP) in patients with cirrhosis: relation to cardiovascular dysfunction and severity of disease. Gut.

[19] Somani PO, Contractor Q, Chaurasia AS, Rathi PM (2014). Diastolic dysfunction characterizes cirrhotic cardiomyopathy. Indian Heart J.

[20] Alexander J, Mishra P, Desai N, Ambadekar S, Gala B, Sawant P (2007). Cirrhotic cardiomyopathy: Indian scenario. J Gastroenterol Hepatol.

[21] Tsutsui JM, Mukherjee S, Elhendy A (2006). Value of dobutamine stress myocardial contrast perfusion echocardiography in patients with advanced liver disease. Liver Transpl.

[22] Coletta C, Galati A, Ricci R, Sestili A, Guagnozzi G, Re F, Ceci V (1997). Prognostic value of left ventricular volume response during dobutamine stress echocardiography. Eur Heart J.

[23] Wong F, Girgrah N, Graba J, Allidina Y, Liu P, Blendis L (2001). The cardiac response to exercise in cirrhosis. Gut.

[24] Grose RD, Nolan J, Dillon JF (1995). Exercise-induced left ventricular dysfunction in alcoholic and non-alcoholic cirrhosis. J Hepatol.

